# F59. VISUALIZING MENTAL REPRESENTATION OF TRUSTWORTHY FACES IN SCHIZOPHRENIA

**DOI:** 10.1093/schbul/sby017.590

**Published:** 2018-04-01

**Authors:** Neeltje Van Haren, Loek Brinkman, Henk Aarts, Ron Dotsch

**Affiliations:** 1 University Medical Centre Utrecht, Brain Centre Rudolf Magnus; 2 Utrecht University

## Abstract

**Background:**

The ability to perceive, recognize and process own and others’ emotions is crucial for efficient and effective social communication. Many different tasks have been used to investigate impairments herein in patients with schizophrenia. Evidence suggests that perception, discrimination and recognition of affective facial expressions are impaired in schizophrenia patients. Importantly, not everyone may interpret the same facial expression similarly. People match their internal representation of specific facial expressions to perceived faces and variation in these internal representations may result in a distortion of social reality. The impairments in face and/or emotion processing and the bias toward a more negative experience may be causally related to degradation of the internal representation itself or to disturbances in the higher-order evaluation of visual input against functionally intact internal representations. In an attempt to develop ways of visualizing an individuals’ internal representation of an emotional face on a computer screen, we set out to visualize the representation of a male and a female face.

**Methods:**

We use a data-driven technique, i.e. reverse correlation image classification (RCIC), which makes it possible to visualize internal representations of faces on computer screens. Participants judge noisy images of faces that are created by superimposing random noise on a single constant base face. The random noise distorts the base face at the pixel level, generating facial variation across stimuli that is fully unconstrained and unaffected by researchers’ a priori expectations. The participants’ responses to a large number of faces are used to model the facial information that was idiosyncratically diagnostic for the judgments. This analysis yields a classification image (CI) for each participant, which visualizes the facial characteristics that drive judgments of emotional expressions (i.e., their internal representation).

We introduce an objective metric, i.e. infoVal, using gender as proof-of-principle. infoVal quantifies the probability that an observed CI was not generated by a random process and is equivalent to a modified z score. First, we test the association between infoVal and more common markers of data quality, i.e. the subjective recognizability, objective discriminability and test-retest reliability of CIs (convergent validity). Second, we use RCIC to investigate and reconstruct the mental representation of trustworthiness as expressed on the face in 32 patients with schizophrenia and 39 controls.

**Results:**

Subjective ratings showed that male and female CIs were more strongly associated with masculinity and femininity, respectively, when infoVal scores where high (p<.001). Second, infoVal scores were highly correlated with test-retest reliability, i.e., higher scores corresponded with higher test-retest reliability (p<1x10-13).

Preliminary analyses of the RCIC task on the internal representation of trustworthy and untrustworthy faces showed that both patients and controls are capable of performing the task adequately. Data-driven multidimensional scaling of the classification images implicate 3 clusters of images, reflecting untrustworthy, neutral, and trustworthy faces. These first analyses suggest that there is no evidence for differences in internal representation of (un)trustworthy faces between patients and controls.

**Discussion:**

We showed how infoVal scores can facilitate the interpretation of CIs. This opens the way to comprehensively investigate internal representations of emotional faces in patients with schizophrenia.

